# A multi-scale mathematical framework for modelling the dynamic nature of autism spectrum disorder symptoms: integrating predictive coding, information theory, and network principles

**DOI:** 10.3389/fpsyt.2026.1787120

**Published:** 2026-04-22

**Authors:** Marios Adamou, Athanasios Kehagias, Grigoris Antoniou

**Affiliations:** 1 School of Human and Health Sciences, University of Huddersfield, Huddersfield, United Kingdom; 2 Aristoteleio Panepistemio Thessalonikes, Thessaloniki, Greece; 3 Leeds Beckett University, Leeds, United Kingdom

**Keywords:** autism spectrum disorder, information theory, mathematical modelling, network neuroscience, predictive coding, repetitive behaviour, restricted interests, sensory processing

## Abstract

**Background:**

Autism Spectrum Disorder (ASD) is characterised by persistent deficits in social communication and interaction alongside restricted, repetitive patterns of behaviour, interests, or activities. Standard diagnostic criteria provide static descriptions, failing to capture the dynamic variability of these symptoms across contexts and developmental trajectories. Furthermore, current descriptions often lack mechanistic explanations for why these symptom fluctuations occur. There is a need for dynamic, theory-driven models that bridge neurobiological mechanisms with observable clinical phenomena.

**Objective:**

This study aimed to develop and present a set of interpretable mathematical models representing the dynamic, context-dependent nature of the core symptoms of ASD, explicitly grounded in established neuropsychological theories including Predictive Coding, Information Theory, and Network Neuroscience principles.

**Methods:**

Mathematical models were theoretically derived using algebraic and differential equations to represent hypothetical symptom dynamics across both diagnostic domains. Social reciprocity was modelled using modulated exponential decay functions derived from executive function and predictive processing theories. Nonverbal communication was represented by weighted summation reflecting multi-channel integration demands. Relationship development was modelled using sigmoid growth functions incorporating social motivation theory. Stereotyped movements were represented by sinusoidal functions conceptualised as homeostatic entropy-reduction mechanisms. Insistence on sameness was modelled using sigmoid preference functions explicitly derived from Bayesian precision-weighting of prediction errors. Restricted interests were represented by exponential functions reflecting atypical reward processing. Sensory sensitivities were modelled using integral functions representing habituation failure due to excitation-inhibition imbalance. No empirical data were collected; parameter ranges were derived from theoretical considerations and existing literature.

**Results:**

The study produced theoretical mathematical equations that quantify the temporal dynamics and contextual modulation for each core symptom domain. These equations provide a formalised representation grounded in mechanistic principles: how social reciprocity would decay due to executive and predictive processing load, how nonverbal communication effectiveness would depend on multi-channel integration capacity, how relationships would develop under constraints of social motivation, how repetitive behaviours would fluctuate to regulate environmental entropy, how preference for sameness would emerge from aberrant Bayesian precision, how restricted interests would persist due to heightened reward salience, and how sensory sensitivities would accumulate due to habituation failure. These models generate testable quantitative predictions about symptom patterns and intervention effects.

**Conclusion:**

The proposed mathematical models offer a novel, quantitative framework for understanding and representing the dynamic nature of ASD symptoms. By integrating mechanistic neurobiological theories with dynamic mathematical formulations, these interpretable models provide a potential advance over static descriptions and may facilitate improved clinical assessment, identification of intervention targets at the parameter level, personalised treatment strategies, and targeted research into the mechanisms underlying ASD. The framework respects neurodiversity by conceptualising many symptoms as adaptive responses to neurobiological differences rather than mere deficits. Empirical validation is required to test model predictions, estimate actual parameter values from clinical populations, and establish clinical utility.

## Introduction

Autism Spectrum Disorder (ASD) is a neurodevelopmental condition affecting millions of people globally and contributing significantly to lifelong challenges in social, educational, and occupational functioning. Current estimates suggest approximately 1 in 100 children worldwide are diagnosed with the condition (1). This prevalence reflects improved diagnostic recognition, broadened diagnostic criteria (2), and greater awareness amongst the general population (3).

ASD manifests early in development, typically before three years of age, and persists throughout the lifespan, though symptom presentation evolves considerably across developmental stages. The condition is characterised by two primary symptom domains: persistent deficits in social communication and social interaction across multiple contexts, and restricted, repetitive patterns of behaviour, interests, or activities (4). These core features present a significant burden on people, families, and healthcare systems (5, 6). The condition has complex aetiologies involving genetic, neurobiological, and environmental factors (7). Recent advances in neuroimaging, genetics, and computational neuroscience have provided insights into underlying mechanisms, revealing alterations in brain connectivity, synaptic function, neural development, and information processing (8, 9). The considerable variability in symptom profiles and the presence of common co-occurring conditions (10) has prompted researchers and clinicians to explore more personalised approaches to diagnosis and intervention (11).

ASD manifests through a heterogeneous range of symptoms, which vary considerably both between people and within people across different contexts and developmental periods. Core symptoms interact dynamically (12), with symptoms fluctuating based on social demands, environmental predictability, sensory characteristics, and internal factors such as anxiety, arousal, or cognitive load (13). For example, a person with ASD may demonstrate relatively good social functioning in highly structured, predictable environments but experience significant difficulties in novel or complex social situations. Similarly, restricted interests are context-dependent, with people showing intensified engagement during periods of stress or reduced external demands. Research has shown that people with ASD exhibit distinct patterns of social attention (14), with reduced spontaneous attention to social stimuli and altered processing of social information (15). Meta-analytic evidence reveals medium to large effect sizes for differences in social orienting and joint attention between people with ASD and typically developing peers (16), indicating fundamental differences in the allocation of attentional resources to social versus non-social information, reflecting underlying neurobiological variations in reward processing and social motivation systems.

The current diagnostic frameworks, such as the DSM-5 (4) and ICD-11 (17), provide descriptive symptom criteria for ASD and offer only a static representation of symptoms. The evolving nature of core symptoms over time, particularly in response to developmental changes, learning, and environmental modifications, is inadequately captured by these static snapshots. Furthermore, whilst these diagnostic frameworks describe what symptoms look like, they do not explain why these symptoms occur or why they fluctuate in the observed patterns in different environments. This gap has led to growing interest in using mathematical modelling to represent the progression, interaction, and variability of symptoms over time, grounded in mechanistic neurobiological theories.

Mathematical models have been applied successfully in various areas of healthcare and neurodevelopmental research to represent complex processes, including disease progression, learning trajectories, and behavioural prediction and in neurodevelopmental disorders, more recently in ADHD (18). In neurodevelopmental conditions, models based on Bayesian networks, differential equations, and machine learning techniques have been used to classify conditions or predict developmental outcomes (19–21). Recent advances in computational neuroscience and developmental modelling have demonstrated the utility of dynamic systems approaches in characterising neurodevelopmental conditions (22–24).

Whilst machine learning models such as Support Vector Machines and deep neural networks have demonstrated high accuracy in ASD classification, they often suffer from limited interpretability that constrains clinical utility (25). Similarly, whilst growth curve models and mixed-effects models are effective for longitudinal data (26, 27), they may lack the flexibility to represent the specific neuropsychological and developmental mechanisms inherent to ASD.

Given these limitations, there is a need for mathematical models that can directly represent the core symptoms of ASD and characterise their evolution across time and context whilst being explicitly grounded in neurobiological theory. To achieve this, any meaningful mathematical model must be anchored in contemporary mechanistic explanations of autism. Numerous theoretical frameworks have been proposed to explain ASD, including the Theory of Mind deficit hypothesis (28), Executive Dysfunction theory (29), Weak Central Coherence theory (30), and the Intense World theory (31). However, these frameworks primarily offer cognitive or phenomenological accounts that are difficult to operationalise mathematically with precision.

We have therefore selected three frameworks: predictive coding, information theory, and network neuroscience based on five specific criteria that would allow robust mathematical modelling. First, mathematical tractability: each framework provides quantifiable constructs that can be directly translated into mathematical parameters (prediction error precision-weighting, entropy measures, and connectivity coefficients respectively). Second, mechanistic depth: these frameworks offer explanations that link neurobiological processes to observable behaviour, rather than describing symptoms at a purely psychological level. Third, empirical validation: each framework has substantial empirical support from neuroimaging, behavioural, and computational studies in ASD populations (32–38). Fourth, complementarity: rather than competing explanations, these three frameworks address different but interconnected aspects of the same underlying processes. For example, predictive coding explains how the brain processes information, information theory quantifies the uncertainty that the brain must manage, and network neuroscience describes the neural architecture that constrains information processing. Fifth, established computational formalism: these frameworks have already demonstrated utility in computational psychiatry more broadly (39–41) providing validated mathematical foundations that can be adapted to ASD-specific phenomena.

In contrast, whilst frameworks such as Theory of Mind deficits remain clinically important for understanding social cognition in ASD, they describe what is impaired without specifying the computational mechanisms underlying that impairment. Executive Dysfunction theory similarly describes functional consequences without providing a formal account of the neurobiological processes involved. Weak Central Coherence theory offers valuable insights into perceptual processing style but lacks the mathematical precision required for dynamic systems modelling. The Intense World theory, whilst addressing sensory and emotional aspects of autism, overlaps substantially with predictive coding accounts but with less developed computational formalisation (32).

Thus, our approach integrates three theoretical frameworks that provide the necessary foundation for mathematically rigorous, mechanistically grounded, and empirically testable models of ASD symptom dynamics.

### The Bayesian brain and predictive coding framework

The predictive coding account of autism proposes that the autistic brain differs fundamentally in how it processes prediction errors, the mismatches between what is expected and what actually occurs (42). According to this framework, neurotypical people assign flexible precision-weighting to prediction errors, allowing them to ignore minor environmental variations whilst attending to genuinely novel or important changes. In autism, this precision-weighting is hypothesised to be aberrant, with excessive precision assigned to low-level sensory prediction errors and potentially reduced precision assigned to higher-level social predictions (32).

This aberrant precision-weighting has cascading consequences. Overweighting of minor sensory prediction errors leads to a world that feels intensely unpredictable and overwhelming, contributing to sensory sensitivities and insistence on sameness. Underweighting of social prediction errors may contribute to difficulties extracting social meaning and regulating social behaviour. The predictive coding framework thus provides a unifying mechanistic explanation for diverse ASD symptoms (39).

### Information theory and entropy reduction

Information theory, originally developed for communication systems, provides a useful tools for understanding uncertainty and predictability (43). Entropy, in this framework, quantifies the degree of unpredictability or disorder in a system. High entropy environments are those where many different states are equally probable, making prediction difficult. Low entropy environments are highly predictable.

Research suggests that people with ASD may experience the environment as having higher entropy than neurotypical people, due to difficulties with prediction and pattern extraction (34). Repetitive behaviours can be conceptualised as entropy-reduction mechanisms by generating highly predictable motor outputs or maintaining highly predictable routines, people create islands of low entropy in an otherwise unpredictable world (44). This framework reframes stereotyped behaviours not as meaningless motor errors but as functional, adaptive responses to an overwhelming sensory and social environment.

### Network neuroscience and connectivity patterns

Network neuroscience examines the brain as a complex network of interconnected regions (45). Research consistently demonstrates atypical connectivity patterns in ASD, characterised by local over-connectivity (increased connections within specific brain regions) but long-range under-connectivity (reduced connections between distant brain regions) (46, 47). This connectivity pattern has functional consequences: processes requiring integration of information across distributed brain regions (such as complex social cognition, language comprehension, or executive function) become metabolically expensive and cognitively demanding (48).

This network architecture helps explain why social interaction, which requires rapid integration of face processing, language comprehension, theory of mind, and executive control across distant brain regions, is particularly demanding for people with ASD and leads to observable fatigue effects (49). It also explains why focused attention on restricted interests, which may engage more localised neural circuits, can be sustained more easily.

### Integrating theoretical frameworks with mathematical models

By focusing on the core symptom domains of ASD such as social communication and interaction deficits and restricted, repetitive behaviours, we aim to create models that capture the temporal and contextual dynamics of these features whilst being explicitly derived from these mechanistic theories. The goal is to provide a framework that can enhance both diagnostic precision and intervention personalisation, offering clinicians and researchers tools to characterise symptom trajectories and tailor support to individual needs. Importantly, by grounding symptoms in adaptive responses to neurobiological differences, the framework adopts a neurodiversity-affirming perspective that respects the internal logic of autistic experience.

## Methodology

### Symptom selection and rationale

The descriptions of ASD symptoms are outlined in the DSM-5 (4). The diagnostic criteria for ASD include symptoms across two primary domains: (A) persistent deficits in social communication and social interaction across multiple contexts, and (B) restricted, repetitive patterns of behaviour, interests, or activities. Not all symptoms described in the DSM-5 can be readily mathematically modelled due to their qualitative or highly context-specific nature.

For ASD, we focused on core, measurable symptoms across both diagnostic domains: social reciprocity, nonverbal communication, relationship development and maintenance, stereotyped or repetitive movements, insistence on sameness, restricted interests, and sensory sensitivities. These symptoms were selected because they are central to the condition’s clinical presentation and have been extensively studied in developmental and cognitive neuroscience research, allowing us to draw upon empirical data and established theories to guide our modelling choices.

For each symptom domain, we identify the relevant mechanistic theory or theories, derive the mathematical form from these theories, and specify parameters that can serve as intervention targets.

### Approach to model development

For each symptom domain, we followed a theory-driven process to develop mathematical models. This process unfolded in five stages. First, we conducted a review of relevant theoretical frameworks, drawing from predictive coding, information theory, and network neuroscience. Second, we identified neuropsychological mechanisms that link neurobiological processes to observable behaviour. Third, we translated these mechanisms into mathematical representations, generating quantifiable parameters. Fourth, we clarified how each parameter should be interpreted to ensure clinical relevance. Fifth, we pinpointed intervention targets at the parameter level. Each model was designed to capture symptoms as dynamic processes shaped by environmental, cognitive, and neurobiological influences rather than as fixed traits. The resulting equations combine mechanistic insight with empirical measurability, offering both conceptual rigour and clinical utility.

### State variable definitions and measurement mapping

To ensure that the mathematical formulations map cleanly to measurable outcomes, we define each state variable in terms of its intended operationalisation. **Table 1** specifies whether each variable represents observable frequency, clinician-rated severity, self-reported distress, or functional impairment, together with the proposed measurement source. This mapping supports future empirical validation by clarifying the level of analysis at which each equation is intended to operate.

**Table 1:** State variable definitions and measurement mapping.

State variable	Definition	Measurement operationalization	Proposed source
R(t)	Social reciprocity level at time t	Frequency and quality of reciprocal social behaviours	Structured observation; clinician rating (e.g., ADOS-2)
N	Nonverbal communication effectiveness	Multi-channel nonverbal cue production and comprehension	Structured observation; video-coded interaction
C(t)	Cumulative relationship quality at time t	Self- or informant-reported relationship depth and number	Self-report questionnaire; informant interview
M(t)	Repetitive movement frequency/intensity at time t	Observable motor stereotypy frequency and amplitude	Actigraphy; observer coding; EMA self-report
S(t)	Distress from routine deviation at time t	Self-reported distress or observer-rated dysregulation	EMA distress ratings; clinician-rated behavioural observation
Lambda_s(t)	Concurrent sensory processing load at time t (instantaneous; used in beta_eff)	Real-time sensory load index derived from environmental characteristics	EMA sensory ratings; environmental sensor data; clinician-rated context
I(t)	Restricted interest engagement intensity at time t	Time allocation and engagement depth with preferred topics	EMA time-use diary; observer-coded engagement
P_total(t)	Cumulative sensory load at time t	Self-reported sensory discomfort or physiological arousal	EMA sensory ratings; skin conductance; heart rate variability

## Results

The following sections present mathematically derived models of core ASD symptoms, grounded in predictive coding, information theory, and network neuroscience. These are theoretical models that generate quantitative predictions and no empirical data were collected in this study. Parameter values presented (e.g., “typical: β_base ≈ 0.1-0.5 hour^-^¹”) represent theoretically plausible ranges derived from underlying neurobiological theories and existing literature, not from experimental measurements. Numerical examples illustrate hypothetical model predictions. Future empirical work will be required to validate these models, estimate actual parameter values from clinical populations, and test predictions against observed data.

It is important to emphasise that the mathematical sophistication of the formulations that follow should not be mistaken for empirical confirmation. The equations are intended as formal representations of theoretically grounded hypotheses, designed to generate testable predictions and to identify specific parameters as potential intervention targets. Their value at this stage lies in their capacity to move beyond static diagnostic descriptions toward dynamic, mechanistic, and falsifiable accounts of symptom expression, not in any claim to validated precision. The framework is presented as a foundation for empirical investigation, and all parameter values, functional forms, and cross-domain couplings require rigorous testing against observed data before clinical application. A comprehensive reference for all symbols, parameters, and their units used throughout the mathematical models is provided in **Table 2**.

**Table 2:** Comprehensive notation reference.

Symbol	Definition	Units	Section
R(t)	Social reciprocity level	Dimensionless (0–1 normalised)	Social Reciprocity
R_0	Initial social reciprocity at interaction onset	Dimensionless	Social Reciprocity
alpha	Individual baseline social capacity coefficient	Dimensionless	Social Reciprocity
beta_base	Baseline executive resource depletion rate	hour^-1	Social Reciprocity
beta_eff	Effective depletion rate (modulated)	hour^-1	Social Reciprocity
k_E, k_Lambda, k_P	Sensitivity coefficients (executive, sensory, predictive load)	Dimensionless	Social Reciprocity
k_M, k_Pr, k_I	Sensitivity coefficients (motivation, predictability, interest)	Dimensionless	Social Reciprocity
Pi_load(t)	Predictive processing load	Dimensionless index	Social Reciprocity
Lambda_s(t)	Concurrent sensory processing load (instantaneous; distinct from S_k and P_total)	Dimensionless index	Social Reciprocity
N	Nonverbal communication effectiveness	Dimensionless composite	Nonverbal Communication
w_i, eta_i	Channel weight and processing efficiency	Dimensionless (0 to 1)	Nonverbal Communication
C(t)	Cumulative relationship quality	Dimensionless composite	Relationships
a_j, k_j, t_j	Sigmoid parameters (asymptote, growth rate, inflection)	Dimensionless; month^-1; months	Relationships
M_soc, R_sal	Social motivation; reward salience	Dimensionless ratios	Relationships
M(t)	Repetitive movement intensity	Frequency index	Repetitive Movements
Gamma_eff, Gamma_max	Effective and maximum oscillation amplitude	Frequency units	Repetitive Movements
omega	Angular frequency of oscillatory cycle	radians hour^-1	Repetitive Movements
H_env(t)	Environmental entropy	Bits (information-theoretic)	Repetitive Movements
k_H	Half-saturation constant for entropy effect	Bits	Repetitive Movements
Phi_alt(t)	Availability of alternative regulatory behaviours	Dimensionless index	Repetitive Movements
S_k	Distress from deviation in routine k	Dimensionless (0 to s_k)	Insistence on Sameness
pi_k	Bayesian precision assigned to prediction errors for routine k	Dimensionless	Insistence on Sameness
theta_k	Tolerance threshold for routine k	Context-dependent deviation units	Insistence on Sameness
s_k	Maximum distress for routine k	Dimensionless severity	Insistence on Sameness
r_k	Response steepness (precision-weighted)	Deviation units^-1	Insistence on Sameness
I(t)	Restricted interest engagement intensity	Dimensionless engagement index	Restricted Interests
I_0	Baseline persistent engagement floor	Dimensionless	Restricted Interests
g	Number of distinct restricted interests	Count	Restricted Interests
b_l, lambda_l	Initial intensity and decay rate for interest l	Dimensionless; month^-1	Restricted Interests
R_int,l, P_acc,l	Reward value and prediction accuracy for interest l	Dimensionless ratios	Restricted Interests
P(s)	Cumulative sensory impact from stimulus s	Dimensionless cumulative index	Sensory Sensitivities
sigma_s	Intensity of sensitivity to stimulus s	Dimensionless sensitivity index (hour^-1)	Sensory Sensitivities
delta_s	Habituation (+) or sensitisation (-) rate	hour^-1	Sensory Sensitivities
Xi(t)	Arousal/anxiety level at time t	Dimensionless index	Sensory Sensitivities
k_sens	Sensitisation coefficient linking arousal to habituation impairment	Dimensionless	Sensory Sensitivities
delta_base,s	Baseline habituation rate for stimulus s	hour^-1	Sensory Sensitivities
phi	Phase shift of oscillatory cycle	Radians	Repetitive Movements
GABA_func/GABA_norm	GABAergic function ratio	Dimensionless ratio	Sensory Sensitivities
A(t)	Composite ASD symptom measure	Dimensionless (normalised composite)	Composite Function
alpha_1-3, beta_1-4	Domain weighting coefficients	Dimensionless (sum to 1)	Composite Function

### ASD: Mathematical models of core symptoms

#### Domain A: social communication and social interaction

##### Social reciprocity: resource depletion through executive and predictive processing demands

###### Theoretical framework

Social reciprocity refers to the natural back-and-forth flow of social interaction, involving the mutual exchange of social behaviours, emotional expressions, and communicative acts. It encompasses the ability to initiate and respond to social overtures, share emotional states, and engage in coordinated social exchanges (50). Social reciprocity forms the foundation for successful social relationships and is essential for typical social development.

In typical development, social reciprocity emerges early in infancy through proto-conversations, shared gaze, and affective synchrony between infant and caregiver (51). As children develop, social reciprocity becomes increasingly sophisticated, incorporating verbal exchanges, perspective-taking, and complex social coordination. This developmental progression reflects the maturation of neural systems supporting social cognition, including the mirror neuron system, theory of mind networks, and reward processing circuits (52).

People with ASD demonstrate consistent difficulties with social reciprocity across the lifespan. These difficulties manifest as reduced initiation of social interactions, diminished responsiveness to others’ social bids, difficulties maintaining conversational flow, and challenges with the timing and coordination of social exchanges (4). Research indicates that deficits in social reciprocity in ASD are associated with differences in neural systems supporting social motivation, social reward processing, and social perception (53, 54).

The temporal dynamics of social reciprocity in ASD reveal particular patterns. During social interactions, people with ASD often show declining engagement over time, particularly in interactions lacking clear structure or alignment with their interests (55). This declining responsiveness requires mechanistic explanation.

###### Neuropsychological mechanisms

Social interaction requires the rapid integration of multiple processes distributed across distant brain regions: face processing (fusiform gyrus, superior temporal sulcus), language comprehension (temporal and frontal language areas), theory of mind (medial prefrontal cortex, temporoparietal junction), executive control (dorsolateral prefrontal cortex), and reward processing (striatum, ventromedial prefrontal cortex (56).

Network neuroscience research demonstrates that ASD is characterised by reduced long-range connectivity between these distributed regions (46, 47) Consequently, social processing requires compensatory recruitment of executive functions such as working memory to maintain conversation threads, inhibitory control to regulate responses, cognitive flexibility to adapt to changing social contexts, and sustained attention to monitor multiple social cues simultaneously (29, 57).

From predictive coding theory, social interaction also requires continuous generation and updating of predictions about others’ intentions, emotional states, and likely responses (58). When these predictions are less accurate (as hypothesised in ASD due to reduced social experience and atypical social reward processing), the prediction errors are larger and require more cognitive resources to resolve (59).

These executive and predictive processing demands function as a depletable cognitive resource. Research on ego depletion and cognitive fatigue demonstrates that sustained engagement of executive functions leads to performance decrements over time (60). In ASD, where social processing inherently requires greater executive engagement due to reduced automaticity, this depletion occurs more rapidly.

###### Mathematical representation

We model social reciprocity as an exponentially decaying resource, modulated by factors that increase or decrease the rate of depletion:

R(t) = αR_0_ exp(-β_eff · t).

where the effective decay rate is:

β_eff = β_base × [(1 + k_E · E(t) + k_Λ · Λ_s(t) + k_P · Π_load(t))/(1 + k_M · M + k_Pr · Pr + k_I · I)].

Parameters:

- R(t): Level of social reciprocity at time t.

- R_0_: Initial level of social reciprocity at interaction onset.

- α: Individual baseline social capacity coefficient.

- β_base: Baseline executive resource depletion rate.

- β_eff: Effective depletion rate modulated by contextual factors.

Factors increasing depletion rate (numerator):

- E(t): Executive load (working memory, inhibition, flexibility demands).

- Λ_s(t): Concurrent sensory processing load (instantaneous; distinct from S_k sameness-distress and P_total cumulative load).

- Π_load(t): Predictive processing load (magnitude and frequency of prediction errors).

- k_E, k_Λ, k_P: Individual sensitivity coefficients for each factor.

Factors decreasing depletion rate (denominator):

- M: Social motivation (intrinsic reward value of social interaction).

- Pr: Environmental predictability (reduced prediction errors).

- I: Interest alignment (interaction relates to restricted interests).

- k_M, k_Pr, k_I: Individual sensitivity coefficients for each protective factor.

###### Interpretation

This formulation captures several theoretically grounded features. The exponential decay term represents resource depletion, aligning with research on cognitive fatigue. The parameter β_base reflects stable individual differences in executive function capacity, which are associated with prefrontal efficiency. Executive load (E) accelerates depletion, helping explain why complex or demanding social contexts are more tiring. Sensory load (Λ_s) similarly increases depletion, consistent with evidence on sensory–cognitive interference. Predictive processing load (Π_load) contributes additional strain, fitting with predictive coding accounts of cognitive effort. In contrast, social motivation (M) reduces depletion by providing intrinsic reward, a pattern supported by social motivation theory (53). Predictability (Pr) also reduces depletion by minimising prediction errors, while interest alignment (I) lowers depletion by engaging inherently rewarding neural circuits (61).

Taken together, the model moves beyond descriptive accounts and offers a mechanistic explanation for several well−observed phenomena. Social interaction becomes especially fatiguing in noisy environments because sensory load is high. Novel social situations are more demanding than familiar ones because predictability is low. Many autistic individuals can sustain interaction for longer when discussing special interests because interest alignment is high. And the need for recovery time after social demands follows naturally from the model’s framing of resource restoration.

###### Clinical and intervention implications

Because the model specifies parameters at a granular level, it supports the design of targeted therapeutic interventions. When β(b_ase_) is high, interventions that strengthen executive function are likely to be beneficial. When k_(_Λ_)_ is elevated, providing sensory accommodations during social interaction becomes important. Low social motivation M suggests a need to identify or cultivate intrinsically rewarding social contexts. High k_(_Pr_)_ indicates that increasing environmental structure and predictability may reduce depletion. Finally, when k_(_I_)_ is high, facilitating social interactions that align with an individual’s interests can meaningfully support sustained engagement.

##### Nonverbal communication: multi-channel integration demands

###### Theoretical framework

Nonverbal communication encompasses the transmission and reception of information through channels other than spoken or written language. This includes facial expressions, eye gaze, body posture, gestures, proxemics, paralinguistic features, and other nonverbal signals (62). Nonverbal communication serves multiple functions in social interaction, including conveying emotional states, regulating conversational flow, emphasising or modifying verbal messages, and establishing social connection.

In typical development, nonverbal communication skills emerge early and develop rapidly during infancy and early childhood. Infants demonstrate sensitivity to facial expressions, vocal prosody, and gestural communication from birth, and begin producing their own nonverbal signals within the first months of life (63). The integration and coordination of multiple nonverbal channels develop throughout childhood and adolescence, becoming increasingly sophisticated and context appropriate.

People with ASD show distinctive patterns in nonverbal communication across multiple domains. Reduced or atypical eye contact is one of the earliest and most consistent features (64). Facial expressions may be less frequent, less varied, or less well-coordinated with social context (65). Gestures, particularly declarative gestures used to share interest or direct attention, are often reduced or delayed in emergence (66). The deficits in nonverbal communication in ASD are not uniform across all channels or contexts, with considerable heterogeneity across people (67).

###### Neuropsychological mechanisms

Effective nonverbal communication requires the simultaneous processing and integration of multiple sensory channels. From an information−processing perspective, this multi−channel integration places demands on several interconnected systems. It relies on parallel processing capacity, because multiple streams of information must be monitored at the same time. It also depends on temporal integration, as signals across channels such as facial expressions and vocal tone which must be synchronised to maintain face−voice congruence. The interpretation of these signals further requires contextual modulation, since the meaning of nonverbal cues shifts depending on the social context in which they occur. Finally, the production of coordinated nonverbal signals depends on fine motor control, making motor coordination an essential component of effective nonverbal communication.

Network neuroscience research indicates that multi-sensory integration relies on long-range connectivity between sensory processing regions and higher-order integration areas (68). The reduced long-range connectivity characteristic of ASD (46) makes this integration process less efficient, requiring compensatory resources.

Additionally, reduced attention to social stimuli in ASD (16) means that certain nonverbal channels (particularly eye gaze) receive reduced processing, creating an asymmetry in channel effectiveness.

###### Mathematical representation

We model nonverbal communication effectiveness as a weighted summation across channels, where weights reflect both the normative importance of each channel and individual processing efficiency:

N = Σ_i_^n^ w_i_x_i_.

where w_i_ = w_norm,i × η_i.

Parameters:

- N: Overall nonverbal communication effectiveness.

- n: Number of distinct nonverbal communication channels.

- x_i_: Frequency and appropriateness of nonverbal cue i.

- w_i_: Effective weight of channel i.

- w_norm,i: Normative importance of channel i in typical communication.

- η_i: Individual processing efficiency for channel i (0 ≤ η_i ≤ 1).

Channels include:

- x_1_: Eye gaze and gaze following.

- x_2_: Facial expression production and recognition.

- x_3_: Gesture production and comprehension.

- x_4_: Body posture and orientation.

- x_5_: Paralinguistic features (prosody, tone, volume).

- x_6_: Proxemics (use of personal space).

###### Interpretation

This formulation captures several theoretically grounded features of relationship development. The sigmoid form reflects the cumulative nature of relational growth. The parameter a_j_ is reduced when social motivation M_so_c is low, consistent with social motivation theory. The growth rate k_j_ decreases when reward salience R_as_l is low, reflecting slower reinforcement learning, and it is similarly reduced when prediction−error variance P_var_ is high, indicating that greater uncertainty slows the consolidation of relational expectations. Because each relationship emerges within a distinct context, different relationships follow different developmental trajectories, and the summation across all relationships represents an individual’s total level of social integration.

For autistic individuals, this framework highlights characteristic patterns such as reduced a_j_ values (relationships not reaching typical depth), reduced k_j_ values (slower development), and delayed t_j_ values (later formation). It also accounts for fewer total relationships in the summation and greater variability in parameters across relationships, with some developing more typically than others.

###### Clinical and intervention implications

The parameter−level structure supports the design of targeted therapeutic interventions. When M_so_c is low, interventions can focus on identifying or cultivating intrinsically rewarding social contexts. When R_sa_l is low, explicit positive reinforcement can strengthen engagement. High P_var_ suggests the need for increased predictability and structure in social interactions. When k is low, allowing longer timeframes for relationship development becomes essential. The model also underscores the value of supporting relationships aligned with an individual’s interests, where intrinsic M_so_c is naturally higher.

##### Developing and maintaining relationships: social motivation and reward processing

###### Theoretical framework

The capacity to develop and maintain relationships represents a higher-order social ability that integrates multiple social-cognitive skills, including social motivation, social perception, social understanding, and behavioural flexibility (69). Relationships vary in type, depth, and developmental timing, with different relationship types becoming salient at different life stages.

People with ASD experience well-documented difficulties in developing and maintaining relationships across the lifespan (70). Longitudinal research indicates that relationship difficulties persist into adulthood for many people, with lower rates of close friendships, romantic relationships, and independent social participation (71, 72). However, considerable heterogeneity exists, with some people developing satisfying relationships, particularly when environments are supportive and accepting (55).

The development of individual relationships over time often follows a sigmoid pattern in typical development, with an initial period of slow development, followed by more rapid growth as mutual understanding and comfort increase, eventually reaching a plateau representing the established relationship level.

###### Neuropsychological mechanisms

The social motivation theory of autism proposes that reduced social motivation, stemming from atypical reward processing, is a core mechanism underlying social difficulties in ASD (53). Neuroimaging research reveals reduced striatal activation during social reward processing in people with ASD (53, 73). This means that social interactions provide less intrinsic reward, making the investment of effort into relationship development less reinforcing.

From a reinforcement learning perspective, relationship development can be conceptualised as a learning process where positive social experiences reinforce approach behaviours and relationship investment (53). When social rewards are devalued (reduced reward salience) or when social experiences are more unpredictable (higher prediction errors), learning is slower and asymptotic performance is lower.

Additionally, the sigmoid growth pattern reflects the cumulative nature of shared experiences, mutual understanding, and established interaction patterns. In ASD, this growth may be slower (reduced growth rate) due to reduced social motivation, lower (reduced asymptote) due to persistent difficulties, or delayed (later inflection point) due to later development of compensatory strategies.

###### Mathematical representation

We model relationship development using sigmoid growth functions, with parameters explicitly linked to social motivation and reward processing:

C = Σ_j_=_1_^m^ [a_j_/(1 + exp(-k_j_(t - t_j_)))].

where:

a_j_ = a_max,j × (M_soc/M_norm).

k_j_ = k_base,j × (R_sal/R_norm) × (1 - P_var).

Parameters:

- C: Cumulative relationship quality at time t.

- m: Number of relationships.

- a_j_: Maximum depth of relationship j, modulated by social motivation.

- a_max,j: Potential maximum depth (context and relationship type dependent).

- M_soc: Individual social motivation level.

- M_norm: Normative social motivation level.

- k_j_: Growth rate for relationship j, modulated by reward salience and predictability.

- k_base,j: Baseline growth rate (relationship type dependent).

- R_sal: Reward salience (strength of social reward signal).

- R_norm: Normative reward salience.

- P_var: Prediction error variance (uncertainty in social interactions).

- t_j_: Inflection point (time of half-maximum development).

- t: Current time or age.

###### Interpretation

This formulation captures several theoretically grounded features of relationship development. The sigmoid shape represents the cumulative nature of relational growth, where depth increases gradually and then accelerates before stabilising. The parameter a_j_ decreases when social motivation M_so_c is low, aligning with social motivation theory, while the growth rate k_j_ is reduced when reward salience R_sa_l is diminished, reflecting slower reinforcement learning. The same parameter k_j_ is also reduced when prediction−error variance P_var_ is high, indicating that greater uncertainty slows the consolidation of relational expectations. Because different relationships unfold within different contexts and social roles, each one follows its own trajectory. The summation across all relationships then represents an individual’s overall level of social integration.

When applied to autistic social development, the model highlights characteristic patterns such as reduced a_j_ values, meaning relationships may not reach the depth typically observed; reduced k_j_ values, indicating slower development; and delayed t_j_ values, reflecting later formation. It also accounts for the tendency to have fewer total relationships, as well as greater variability in parameters across relationships, with some developing in a more typical manner than others.

###### Clinical and Intervention Implications

Because the model specifies parameters at a fine−grained level, it supports targeted intervention strategies. When social motivation M_so_c is low, interventions can focus on identifying or cultivating intrinsically rewarding social contexts. When reward salience R_sa_l is low, explicit positive reinforcement can help strengthen engagement. High prediction−error variance P_var_ suggests the need for increased predictability and structure in social interactions. When the growth rate k_j_ is low, allowing longer timeframes for relationship development becomes essential. The model also underscores the value of supporting relationships that align with an individual’s interests, where intrinsic M_so_c is naturally higher.

#### Domain B: restricted, repetitive patterns of behaviour, interests, or activities

##### Stereotyped or repetitive movements: homeostatic entropy reduction

###### Theoretical framework

Stereotyped or repetitive movements, often referred to as stereotypies or self-stimulatory behaviours, encompass a range of rhythmic, repetitive, and often seemingly purposeless motor behaviours (74). These behaviours include hand flapping, finger flicking, body rocking, spinning, pacing in patterns, and repetitive manipulation of objects. Whilst some repetitive movements are observed in typical development, particularly in infancy and toddlerhood, the stereotypies seen in ASD tend to be more frequent, more persistent across development, and more likely to occur during various activities and emotional states (75).

The functions of repetitive movements in ASD appear to be multiple and complex. Research suggests these behaviours may serve to regulate arousal, manage sensory input, reduce anxiety, provide predictable sensory feedback, or express emotional states (76, 77). Different repetitive movements may serve different functions for the same individual, and the function may vary across contexts.

The frequency and intensity of repetitive movements in ASD fluctuate based on multiple factors. These behaviours often increase during periods of heightened emotional arousal (whether positive or negative), cognitive demand, environmental unpredictability, or sensory overload (78). Conversely, they may decrease during highly engaging activities aligned with interests, in familiar and comfortable environments, or during activities requiring use of the hands or body in other ways (79).

The temporal pattern of repetitive movements often shows rhythmic, oscillatory characteristics, with behaviours waxing and waning in intensity over time. This cyclical pattern may reflect underlying physiological rhythms, homeostatic regulatory processes, or responses to fluctuating internal and external demands.

###### Neuropsychological mechanisms

From information theory, entropy (H) quantifies the unpredictability or disorder in a system (43). A high-entropy environment is one where many different states are equally probable, making prediction difficult. A low-entropy environment is highly predictable.

Research suggests that people with ASD may experience the environment as having higher entropy than neurotypical individuals (34). This elevated subjective entropy appears to arise from several interacting mechanisms. Difficulties with pattern extraction and prediction, consistent with predictive−coding accounts, make it harder to form stable expectations about incoming sensory information. Heightened sensitivity to environmental variations, driven by aberrant precision−weighting, causes even small changes to be registered as meaningful or salient. Reduced habituation to repetitive stimuli further contributes to this effect, as background noise or recurring sensory input does not fade into irrelevance but instead remains continuously salient. Together, these mechanisms create an environment that feels more unpredictable, information−dense, and harder to filter.

Repetitive movements can be conceptualised as entropy-reduction mechanisms. By generating highly predictable motor outputs with near-zero entropy, people create islands of low entropy in an otherwise unpredictable world (44). The predictable sensory feedback from these movements (proprioceptive, visual, auditory) provides a controllable, low-entropy signal that can be used to regulate arousal and anxiety.

From a homeostatic perspective, the amplitude of repetitive behaviours should increase when environmental entropy is high (to provide greater entropy reduction) and decrease when environmental entropy is low (when less regulation is needed).

###### Mathematical representation

We model repetitive movements as oscillatory behaviours with amplitude modulated by environmental entropy:

M(t) = M_0_ + Γ_eff sin(ωt + φ).

where the effective amplitude is:

Γ_eff = Γ_max × [H_env(t)/(H_env(t) + k_H)] × [1/(1 + k_E · E(t) + k_Φ · Φ_alt(t))].

Parameters:

- M(t): Frequency or intensity of repetitive movements at time t.

- M_0_: Baseline motor activity level.

- Γ_eff: Effective amplitude (degree of variation from baseline).

- Γ_max: Maximum potential amplitude.

- ω: Angular frequency (determining period of oscillatory cycle).

- φ: Phase shift.

- t: Time.

Amplitude modulation factors:

- H_env(t): Environmental entropy (unpredictability) at time t.

- k_H: Half-saturation constant for entropy effect.

- E(t): Engagement level in absorbing activity.

- Φ_alt(t): Availability of alternative regulatory behaviours.

- k_E, k_Φ: Sensitivity coefficients.

###### Interpretation

This formulation captures several theoretically grounded features. The sinusoidal base reflects the rhythmic, oscillatory nature of stereotypies. The effective amplitude A_e_ff increases with environmental entropy H_env_, consistent with entropy−reduction theory, and the Michaelis–Menten form H_env_/(H_env_ + k_h_) introduces saturation, meaning amplitude plateaus at high levels of entropy. The value of A_e_ff decreases with engagement E, which explains why stereotypies diminish during absorbing or highly focused activities, and it also decreases with the availability of alternative regulatory behaviours A_alt_, demonstrating compensation across different self−regulatory strategies. Individual variation in the k parameters reflects heterogeneity in regulatory needs across people and contexts.

When applied to autistic individuals, the model highlights characteristic patterns such as a higher baseline M_0_, indicating more frequent stereotypies at rest; a higher A_max_, reflecting greater maximum intensity; and a lower k_h_, meaning amplitude increases rapidly even with modest increases in environmental entropy. The behaviour itself serves a functional, adaptive purpose, supporting homeostatic regulation rather than representing meaningless or maladaptive action.

This reframing is neurodiversity−affirming as stereotypies are understood as functional responses to neurobiological differences in entropy perception and regulation, rather than as errors to be eliminated.

###### Clinical and intervention implications

Because the model specifies parameters at a fine−grained level, it supports targeted intervention strategies. When H_env_ is consistently high, reducing environmental unpredictability and sensory complexity becomes important. When k_h_ is low, it is essential to recognise heightened sensitivity to environmental changes. Increasing A_alt_ by providing alternative regulatory strategies is more supportive than attempting behavioural extinction. Stereotypies should be supported in contexts where they serve a regulatory function, and reductions should occur indirectly through lowering H_env_, rather than through direct suppression of the behaviour.

###### Note on model selection

The sinusoidal formulation presented here serves as a best first-order approximation that captures the rhythmic, oscillatory quality of many stereotypies (e.g., rocking, hand flapping) and aligns with homeostatic regulation theory. However, many repetitive behaviours manifest as intermittent bursts rather than continuous rhythmic output. A more physiologically realistic representation could employ a hybrid model combining an oscillatory component within bouts and a point-process bout-onset rate modulated by entropy. In such a model, bout onset would follow a non-homogeneous Poisson process with rate function dependent on H_env(t), and within each bout, the behaviour would follow the sinusoidal form presented above. This extension increases the number of parameters (bout duration distribution, inter-bout interval distribution, onset-rate function) and would require high-temporal-resolution observational data for estimation. The sinusoidal model is therefore presented as a tractable baseline, with the hybrid extension identified as a priority for future empirical work.

##### Insistence on sameness: Bayesian precision-weighting of prediction errors

###### Theoretical framework

Insistence on sameness refers to a preference for, and often rigid adherence to, familiar routines, patterns, and environmental configurations (80) (60). This feature of ASD manifests across multiple domains, including daily routines, environmental arrangements, and interaction patterns (4). In typical development, young children often show preferences for routines and may become distressed by changes. However, these preferences typically diminish with age as cognitive flexibility develops. In ASD, preferences for sameness tend to be more intense, more persistent across development, and more resistant to change (61). Disruptions to routines or expectations can result in significant distress, anxiety, or behavioural dysregulation.

The insistence on sameness in ASD has been conceptualised from multiple theoretical perspectives. Cognitive theories emphasise difficulties with executive functions, particularly cognitive flexibility and set-shifting (29). Anxiety-based accounts propose that routines serve an anxiety-reducing function in the face of an unpredictable social and sensory world (81). Neurobiologically, insistence on sameness has been associated with differences in prefrontal-striatal circuits involved in behavioural flexibility and anterior cingulate cortex activation during adaptation to change (82).

###### Neuropsychological mechanisms

The predictive coding framework provides a mechanistic explanation for insistence on sameness (32, 42). According to this theory, the brain continuously generates predictions about upcoming sensory input, actions, and events. When actual input differs from predictions, prediction errors are generated. The influence of these prediction errors on updating future predictions depends on their precision-weighting—highly precise (highly weighted) prediction errors strongly influence belief updating, whilst imprecise (lowly weighted) prediction errors are largely ignored.

In typical people, precision-weighting is context-appropriate. Minor deviations from routine (e.g., a different brand of cereal) generate small prediction errors with low precision-weighting and are easily accommodated. Major deviations (e.g., a fire alarm) generate large prediction errors with high precision-weighting and trigger significant behavioural responses.

The predictive coding account of autism proposes aberrant precision-weighting, particularly overweighting of low-level prediction errors (32). This means that minor environmental changes (small prediction errors) are assigned high precision, making them feel as significant as major changes. This creates a “threshold” effect: once a prediction error exceeds a critical magnitude, it triggers maximal distress because it is weighted as highly significant.

###### Mathematical representation

We model distress in response to routine deviation using a sigmoid function explicitly derived from Bayesian precision-weighting:

S_k = [s_k/(1 + exp(-r_k(|x_k - x̄_k| - θ_k)))].

where the precision-weighting influences the steepness:

r_k = r_base,k × π_k.

Parameters:

- S_k: Distress associated with deviation in routine k.

- s_k: Maximum distress for routine k (importance of routine).

- r_k: Response steepness (precision-weighting parameter).

- r_base,k: Baseline response steepness.

- π_k: Bayesian precision assigned to prediction errors for routine k.

- x_k: Current state of routine k.

- x̄_k: Expected/preferred state of routine k.

- |x_k - x̄_k|: Magnitude of prediction error (deviation from expectation).

- θ_k: Tolerance threshold (deviation magnitude at inflection point).

Total insistence on sameness:

S = Σ_k_=_1_^p^ S_k.

###### Interpretation

This formulation captures several important features grounded in predictive coding theory. The sigmoid shape reflects the threshold−based nature of the distress response, where small deviations below θ_k_ generate minimal distress because prediction errors remain low. Once deviations exceed θ_k_, distress escalates rapidly as the system crosses a perceptual threshold. The steepness parameter r_k_ reflects Bayesian precision π_k_, and when π_k_ is high indicating aberrant precision−weighting, the response curve becomes extremely steep, meaning even small changes produce disproportionately large distress signals. Different routines carry different precision−weightings, creating heterogeneity across contexts. This structure also explains why intervention outcomes depend on the specific parameter configuration rather than any single parameter alone. Gradual exposure may fail for multiple, distinguishable reasons identifiable within the model. If π_k_ remains elevated, the brain continues to interpret even minor changes as critical prediction errors.

For autistic individuals, the model highlights characteristic patterns such as higher π_k_ values, reflecting heightened precision−weighting; lower θ_k_ values, meaning smaller deviations trigger threshold crossing; and higher s_k_ values, indicating greater maximum distress. It also accounts for the presence of more terms in the summation, representing a larger number of routines maintained with high precision.

This formulation aligns with the “Intense World Theory” (31) by showing mathematically how elevated precision−weighting can create a world in which minor changes feel overwhelming or catastrophic.

An important consideration is equifinality because clinically similar distress profiles can arise from distinct parameter configurations. Elevated precision-weighting (high pi_k) steepens the sigmoid response, producing a sharp transition from minimal to maximal distress. However, a constitutionally low tolerance threshold (low theta_k) shifts the inflection point leftward, triggering distress at very small deviations even when precision-weighting is moderate. Similarly, a high maximum distress parameter (high s_k) elevates the asymptote, producing severe dysregulation once any threshold is crossed regardless of steepness. These three routes to clinically similar presentations have distinct intervention implications: high pi_k responds to uncertainty-tolerance training, low theta_k requires graded environmental modification, and high s_k calls for emotion-regulation support. The clinical value of the model therefore lies precisely in its capacity to distinguish between superficially similar presentations with different mechanistic origins, supporting personalised formulation.

###### Clinical and intervention implications

Because the model specifies parameters at a fine−grained level, it supports targeted intervention strategies. When π_k_ is high, interventions should focus on modifying precision−weighting rather than relying solely on exposure, using approaches such as predictability−enhancing strategies or anxiety−reduction techniques. When θ_k_ is low, it is important to recognise that even very small changes can trigger distress, making predictability essential. Gradual exposure alone is insufficient if π_k_ remains elevated. Cognitive−behavioural approaches that strengthen uncertainty tolerance may help reduce π_k_, while environmental modifications that increase predictability can reduce the magnitude of prediction errors.

Because equifinality means that clinically similar distress profiles can arise from distinct parameter configurations, effective clinical formulation requires parameter-level differentiation rather than uniform application of any single intervention strategy.

##### Highly restricted, fixated interests: atypical reward processing

###### Theoretical framework

Highly restricted interests represent intense, focused preoccupation with specific topics, objects, or activities that are abnormal in intensity or focus (4). These interests often dominate the individual’s time, attention, and conversation (83). In typical development, children develop interests and hobbies, which may be intense but typically exist alongside other interests and activities. In ASD, restricted interests tend to be more intense, more narrow in scope, more persistent over time, less likely to align with peer interests, and less responsive to social context (82).

The content of restricted interests varies widely, though certain categories are common (84). The specific interest may change over time, though the pattern of intense, focused engagement typically persists.

###### Neuropsychological mechanisms

Restricted interests in ASD have been closely linked to differences in reward processing and motivation. Neuroimaging studies show heightened striatal activation when individuals engage with preferred interests, indicating that these topics are particularly rewarding (61, 85) Several mechanisms may contribute to this heightened reward response. One possibility is compensatory engagement: when social reward is reduced (54), nonsocial interests may become disproportionately rewarding. Another mechanism involves prediction success, as circumscribed interests often provide domains in which predictions are highly accurate, thereby reducing prediction errors and lowering anxiety. Atypical dopaminergic function may also play a role, altering the salience of specific stimuli and further amplifying reward responses (86).

From a reinforcement−learning perspective, the value V of engaging with an interest reflects both the immediate reward R and the discounted value of future rewards. When an interest offers high immediate reward and low uncertainty meaning prediction accuracy is high and its value remains elevated over time. This helps explain why restricted interests can be so enduring.

The intensity of engagement with restricted interests also fluctuates across time. New interests may evoke particularly strong initial engagement that gradually decreases as the topic becomes more familiar. However, unlike typical interests that often fade substantially, restricted interests in ASD tend to maintain a higher baseline level of engagement over extended periods. This persistence reflects sustained reward salience and the continued predictability and mastery these interests provide.

###### Mathematical representation

We model restricted interest engagement using exponential functions with baseline offset, reflecting sustained reward salience:

I(t) = I_0_ + Σ_l_=_1_^g^ b_l_ exp(-λ_l_t).

where:

b_l_ = b_base,l × (R_int,l/R_norm).

λ_l_ = λ_base,l × (1 - P_acc,l).

Parameters:

- I(t): Overall intensity of restricted interest engagement at time t.

- I_0_: Baseline engagement level (persistent engagement floor).

- g: Number of distinct restricted interests.

- b_l_: Initial intensity of interest l, modulated by reward value.

- b_base,l: Baseline initial intensity.

- R_int,l: Reward value of interest l.

- R_norm: Normative reward value.

- λ_l_: Decay rate for interest l, modulated by prediction accuracy.

- λ_base,l: Baseline decay rate.

- P_acc,l: Prediction accuracy within interest domain l (0 ≤ P_acc,l ≤ 1).

- t: Time since interest l was acquired.

###### Interpretation

This formulation captures several theoretically grounded features. The baseline offset I_0_ represents persistent engagement that does not decay to zero. Higher reward value R_int_,_l_ increases the initial intensity b_l_, while higher prediction accuracy P_a_cc,_l_ reduces the decay rate λ_l_, approaching zero when prediction is highly reliable. Interests that provide both strong reward and high prediction accuracy therefore persist the longest. The model also accommodates multiple concurrent interests, each with its own parameter set, and new interests simply add additional terms to the overall summation.

When applied to autistic individuals, the model highlights characteristic patterns such as elevated I_0_, reflecting a higher baseline level of engagement; higher b_l_, indicating more intense initial engagement; and lower λ_l_, meaning interests decay more slowly and often remain highly persistent. In some cases λ_l_ ≈ 0, producing interests that show virtually no decay over time. Many autistic interests also exhibit a higher R_int_,_i_/R_norm_ ratio, meaning they are disproportionately rewarding, and higher P_a_cc,_l_, reflecting that these interests provide domains of mastery, predictability, and reduced uncertainty.

This formulation reframes restricted interests as adaptive: they offer reliable sources of reward, positive affect, and stability in an unpredictable world shaped by atypical reward processing and heightened sensitivity to uncertainty.

###### Clinical and intervention implications

Because the model specifies parameters at a fine−grained level, it supports targeted intervention strategies. Restricted interests should be recognised as meaningful sources of reward and competence, consistent with neurodiversity−affirming practice. They can be used as motivational tools and as bridges to other activities. When I_0_ or b_l_ is extremely high and contributes to functional impairment, gradual introduction of alternative rewarding activities may help broaden engagement. Social connections can be facilitated through shared interests, leveraging high R_int_,_l_, and interests can be supported as pathways into skills development or vocational opportunities.

##### Hyper- or hypo-reactivity to sensory input: habituation failure and cumulative load

###### Theoretical framework

Sensory sensitivities in ASD encompass both hyper-responsivity (over-responsiveness to sensory input) and hypo-responsivity (under-responsiveness to sensory input), often co-occurring within the same individual across different sensory modalities (87). These sensory differences are recognised in the DSM-5 as core features of ASD and are present in the majority of people with the condition (88).

Hyper-responsivity manifests as excessive or aversive responses to sensory stimuli that others find tolerable. Hypo-responsivity manifests as diminished response to sensory input (89). These sensory differences have significant functional impacts, affecting participation in daily activities, educational settings, social situations, and overall quality of life (90).

The experience of sensory sensitivity is not static but varies with exposure duration, context, and prior sensory experiences. Repeated or prolonged exposure to aversive stimuli can lead to increasing distress (sensitisation), whilst in typical people, brief exposures often lead to habituation (reduced response).

###### Neuropsychological mechanisms

Neurobiological research has revealed differences in sensory processing regions and in connectivity between sensory and higher-order processing areas in ASD (91). Critically, differences in neural habituation to repeated stimuli have been documented (92) Habituation described as the normal reduction in neural and behavioural response to repeated or sustained stimuli appears to be impaired in ASD.

This habituation failure has been linked to excitation-inhibition (E-I) imbalance, particularly reduced GABAergic inhibition (93). GABA is the primary inhibitory neurotransmitter, and it plays important roles in sensory gating and adaptation. Reduced GABA function means that repetitive sensory signals continue to elicit strong responses rather than being filtered or attenuated.

From a mathematical perspective, neural response to a sustained stimulus in typical people follows exponential decay (habituation). In ASD, this decay is greatly reduced or absent (δ ≈ 0), meaning that the response is sustained or may even increase over time (sensitisation, δ < 0).

The cumulative sensory load over an exposure period can be modelled as the integral of instantaneous sensory impact over time. When habituation is absent, this integral grows without bound, eventually exceeding regulatory capacity and triggering meltdown or shutdown.

###### Mathematical representation

We model cumulative sensory impact using an integral formulation with habituation/sensitisation parameter:

P(s) = ∫_0_^T^ σ_s_ exp(-δ_s_t) dt.

Parameters:

- P(s): Cumulative sensory impact from stimulus s over period T.

- T: Duration of exposure.

- σ_s_: Intensity of sensitivity to stimulus s (sensory responsivity).

- δ_s_: Habituation (δ_s_ > 0) or sensitisation (δ_s_ < 0) rate for stimulus s.

- t: Time within exposure period.

For δ_s_ ≠ 0, solving the integral:

P(s) = (σ_s_/δ_s_)[1 - exp(-δ_s_T)].

For δ_s_ = 0 (habituation failure):

P(s) = σ_s_T.

For δ_s_ < 0 (sensitisation):

P(s) = (σ_s_/|δ_s_|)[exp(|δ_s_|T) - 1].

Total cumulative sensory load across modalities:

P_total = Σ_s_ P(s).

Mechanistic parameters:

δ_s_ = δ_base,s × (GABA_func/GABA_norm) - k_sens × Ξ(t).

where:

- δ_base,s: Baseline habituation rate for stimulus s.

- GABA_func: GABAergic function level.

- GABA_norm: Normative GABAergic function.

- k_sens: Sensitisation coefficient.

- Ξ(t): Arousal/anxiety level at time t.

###### Interpretation

This formulation captures several theoretically grounded features. When δ_s_ > 0, representing typical habituation, the function P(s) saturates and shows bounded growth. When δ_s_ ≈ 0, reflecting habituation failure often observed in autistic individuals, P(s) = σ_s_T, producing linear and unbounded accumulation over time. When δ_s_ < 0, the system enters sensitisation, and P(s) grows exponentially, leading to rapid escalation. Reduced GABAergic function decreases δ_s_, impairing habituation, while high anxiety Ξ can push δ_s_ into the negative range, triggering sensitisation. The cumulative nature of P(s) explains delayed meltdowns, where thresholds are crossed only after sustained exposure.

For autistic individuals who experience hyper−responsivity, σ_s_ tends to be elevated, indicating greater sensory sensitivity, while δ_s_ is reduced or near zero, meaning habituation is minimal. As a result, P(s) accumulates rapidly even when σ_s_ is only moderately high, and meltdowns occur when P_total_ exceeds regulatory capacity. In contrast, autistic individuals who experience hypo−responsivity tend to show reduced σ_s_, requiring higher intensity or longer duration of stimulation to reach comparable levels of P(s), and may seek intense sensory input to achieve sufficient activation.

This model explains why seemingly minor sounds, such as a ticking clock, can eventually trigger a meltdown through unbounded accumulation; why sensory environments that feel manageable at first can become overwhelming over time; why removing an individual from sensory input allows recovery as P(s) decays once the stimulus is removed; and why anxiety increases sensory reactivity by reducing δ_s_.

###### Clinical and intervention implications

Because the model specifies parameters at a fine−grained level, it supports targeted intervention strategies. When σ_s_ is high for specific stimuli, environmental accommodations that reduce exposure become essential. When δ_s_ ≈ 0, it is important to recognise that habituation will not occur, making time−limited exposure necessary. When δ_s_ < 0, there is particular urgency to remove the stimulus because escalation becomes exponential. Sensory breaks help reduce P_total_, and teaching self−advocacy skills enables individuals to recognise when P_total_ is approaching threshold. For hypo−responsivity, where σ_s_ is low, providing access to safe sensory input supports regulation.

### Dimensional analysis

To ensure formal consistency, **Table 3** presents a dimensional analysis verifying that all exponent arguments are dimensionless and all additive terms within each equation share compatible dimensions.

**Table 3:** Dimensional analysis of model parameters.

Equation element	Dimensions	Verification
beta_eff * t (exponent in R(t))	[hour^-1] * [hour] = dimensionless	Valid: exponent is dimensionless
k_E * E(t), k_Lambda * Lambda_s(t), k_P * Pi_load(t)	[dimensionless] * [dimensionless] = dimensionless	Valid: additive terms in beta_eff numerator are commensurate
omega * t + phi (argument of sin)	[rad hour^-1] * [hour] + [rad] = radians	Valid: angular argument is dimensionless (radians)
H_env / (H_env + k_H)	[bits] / [bits] = dimensionless	Valid: Michaelis-Menten ratio
r_k * (|x_k - x_bar_k| - theta_k) (exponent in S_k)	[deviation^-1] * [deviation] = dimensionless	Valid: sigmoid exponent is dimensionless
lambda_l * t (exponent in I(t))	[month^-1] * [months] = dimensionless	Valid: exponent is dimensionless
sigma_s * exp(-delta_s * t)	[dimensionless hour^-1] * [dimensionless] = dimensionless hour^-1	Valid: integrand yields dimensionless index upon integration over time
delta_s * t (exponent in P(s))	[hour^-1] * [hour] = dimensionless	Valid: exponent is dimensionless
P(s) = (sigma_s / delta_s) * [1 - exp(-delta_s * T)]	[dimensionless hour^-1] / [hour^-1] = dimensionless	Valid: cumulative impact is dimensionless index
alpha_i * z_i(t) in A(t)	[dimensionless] * [dimensionless] = dimensionless	Valid: all additive terms commensurate after normalisation

All sensitivity coefficients (k_E, k_Lambda, k_P, k_M, k_Pr, k_I, k_H, k_Phi) are dimensionless scaling factors. The weights alpha_i in the composite function are dimensionless following the normalisation scheme described above. Where parameters carry implicit unit assumptions (e.g., sigma_s as a sensitivity index), empirical calibration will assign concrete units based on the chosen measurement instrument.

## Composite function for ASD

### Integrating domains for overall symptom characterisation

A composite function integrating the core symptom domains can be formulated to provide an overall quantitative representation of ASD symptomatology. The expression.

A(t) = α_1_R(t) + α_2_N + α_3_C(t) + β_1_M(t) + β_2_S(t) + β_3_I(t) + β_4_P_total_(t).

captures the combined contribution of social−communication features and restricted, repetitive behaviour features at any given time point.

To ensure commensurability across domains that operate on different scales, each component is normalised to the unit interval [0, 1] before summation, using the transformation z_i(t) = [x_i(t) - x_{i,min}]/[x_{i,max} - x_{i,min}], where x_{i,min} and x_{i,max} represent theoretically derived or empirically estimated bounds for each domain. The composite function is therefore expressed as A(t) = Sum_i alpha_i z_i(t), where the weights alpha_i sum to unity and can be set to reflect diagnostic weighting, functional impact, or individual clinical emphasis.

To clarify the relationship between the two formulations: the original domain-specific weights (alpha_1, alpha_2, alpha_3 for social-communication components and beta_1, beta_2, beta_3, beta_4 for restricted-repetitive behaviour components) are subsumed by the unified weighting scheme w_i (i = 1,…, 7), where each w_i incorporates both the domain-level priority and any scale-correction implied by normalisation. Formally, w_1 = alpha_1, w_2 = alpha_2, w_3 = alpha_3, w_4 = beta_1, w_5 = beta_2, w_6 = beta_3, and w_7 = beta_4, applied to the corresponding normalised components z_i(t). The constraint that the w_i sum to unity ensures commensurability. The earlier alpha/beta formulation is retained for conceptual clarity in linking weights to the two DSM-5 diagnostic domains, whilst the w_i notation is used wherever a single unified index offers a simpler and more coherent approach.

### Note on redundancy

It is acknowledged that partial redundancy exists across domains. For example, cumulative sensory load P_total(t) contributes directly to the composite and also influences social reciprocity R(t) via the sensory load term in beta_eff, creating partial double-counting. Two approaches to managing this are available. The first treats the composite as a descriptive summary rather than a strict causal model, in which partial overlap is acceptable and analogous to correlated subscales in clinical instruments. The second applies a structural equation modelling framework that decomposes shared and unique variance across domains. The latter approach is recommended for future empirical work. The present formulation serves primarily as a conceptual integration rather than a strict additive decomposition.

In this formulation, A(t) represents the overall measure of ASD symptom presence and severity at time t. The coefficients α_1_, α_2_, and α_3_ weight the components associated with the social communication and interaction domain, while β_1_, β_2_, β_3_, and β_4_ weight the components associated with restricted and repetitive behaviours. These coefficients can be determined by considering the relative diagnostic importance of each symptom domain, the individual’s specific symptom profile—since some people show greater severity in particular areas—the developmental stage, which may shift the relevance of certain symptoms over time, and the functional impact each domain has on daily living.

This composite function therefore provides a holistic, quantitative representation of ASD consistent with DSM−5 criteria, integrating both required diagnostic domains into a single framework. Because several components are time−dependent, the formulation also supports the characterisation of symptom trajectories across development and in response to interventions.

## Discussion

### Integrating mechanistic theory with dynamic modelling

This study presents a comprehensive mathematical framework for ASD that integrates dynamic systems theory with established neurobiological mechanisms including Predictive Coding, Information Theory, and Network Neuroscience. By explicitly deriving mathematical formulations from these mechanistic theories, we move beyond purely descriptive models to explanatory models that address both what symptoms look like and why they occur.

### Linking mechanism to behaviour

The mechanistic grounding of the framework provides several important advantages. The formulation of social reciprocity decay through β_e_ff links the decay rate to executive−function demands, predictive−processing load, and limitations in long−range connectivity. This connection explains why social interaction is particularly fatiguing for people with ASD and aligns with clinical observations of burnout and the need for recovery time following social demands (55, 94). The treatment of insistence on sameness through Bayesian precision π_k_ offers a further advantage: by deriving the mechanism directly from predictive−coding theory, the model clarifies why exposure−based interventions often fail when they do not address underlying precision−weighting. If π_k_ remains high, the brain continues to interpret routine changes as critical errors regardless of repeated exposure (32, 95), suggesting that interventions targeting uncertainty tolerance and anxiety reduction may be more effective than forced adaptation.

The framework also reconceptualises repetitive behaviours through the lens of entropic regulation. By modelling stereotypies as functional entropy−reduction mechanisms rather than meaningless motor errors, it provides a neurodiversity−affirming interpretation with significant intervention implications (24).

Instead of attempting behavioural extinction, support should focus on reducing environmental entropy or offering alternative regulatory strategies. Finally, the treatment of sensory sensitivity through the parameter δ ≈ 0 demonstrates mathematically how habituation failure leads to unbounded cumulative load. This explains the delayed onset of sensory meltdowns and highlights the importance of time−limited exposure and appropriate environmental accommodations (91, 96).

### Temporal scales and cross-model coupling

The submodels presented in this framework operate across different characteristic time scales, ranging from seconds (sensory processing) through minutes to hours (social fatigue, repetitive movements) to months and years (relationship development, developmental trajectories). **Table 4** specifies the time scale, time variable, and coupling mechanism for each submodel.

**Table 4:** Temporal scales and cross-model coupling.

Submodel	Characteristic time scale	Time variable	Coupling to other submodels
Sensory Impact P(s)	Seconds to minutes	t within exposure episode	P_total feeds into beta_eff (social reciprocity) via Lambda_s; triggers M(t) via H_env
Social Reciprocity R(t)	Minutes to hours	t within single interaction	Modulated by Lambda_s(t) (sensory load) and Pi_load (prediction errors)
Repetitive Movements M(t)	Minutes to hours	t within environmental context	Driven by H_env; reduced by engagement E(t)
Insistence on Sameness S_k	Minutes-hours (acute); weeks-months (pi_k evolution)	Deviation event time; developmental time for pi_k	pi_k quasi-static within fast submodels
Restricted Interests I(t)	Weeks to months	t since interest acquisition	Provides interest alignment I reducing beta_eff
Relationship Development C(t)	Months to years	t as age or relationship duration	Modulated by M_soc and R_sal (slow-evolving)
Composite A(t) (developmental)	Years	t as developmental age	Summary statistic; not fed back into mechanistic equations

The submodels are hierarchically coupled rather than simultaneously solved. Fast-time-scale processes (sensory load, social fatigue) influence slow-time-scale processes (relationship development, developmental trajectories) via time-averaged or cumulative effects. Slow-time-scale parameters (e.g., pi_k, beta_base) are treated as quasi-static within the time frame of faster submodels. This hierarchical separation of time scales is a standard approach in multi-scale dynamical systems modelling and avoids algebraic loops. The composite function A(t) is not fed back into the mechanistic equations within the same time step; rather, it serves as a summary statistic for clinical characterisation and longitudinal tracking. Where arousal Xi(t) influences delta_s, this is modelled as a lagged effect, with Xi(t) derived from the preceding time period rather than the current instant.

#### Clinical utility and personalised phenotyping

These equations support personalised computational phenotyping by moving beyond categorical diagnosis and instead characterising each individual through their unique parameter profile. One person might show a pattern defined by high β_(_b_ase)_, indicating rapid social fatigue; low π_k_, reflecting relatively good tolerance for change; moderate sensitivity to H_env_, producing moderate levels of stereotypy; and high σ for auditory stimuli, consistent with auditory hyper−responsivity. Another individual might instead show moderate β_(_b_ase)_, suggesting typical social endurance; very high π_k_, leading to extreme distress when routines change; low H_env_ sensitivity, resulting in minimal stereotypy; and low δ across sensory modalities, indicating severe habituation failure.

These distinct parameter signatures allow for more precisely targeted support strategies. For someone with the first profile, interventions would prioritise reducing social demands and providing sensory accommodations. For someone with the second profile, the focus would shift toward increasing environmental predictability and addressing Bayesian precision−weighting through approaches that build uncertainty tolerance. This personalised mapping enables interventions that align closely with each individual’s neurobiological configuration.

#### Parameter−level intervention targets

Within this framework, specific parameters become direct intervention targets. Executive−function−focused approaches aim to influence β_(_b_ase)_, using methods such as working−memory training, strategy instruction, or task simplification. Environmental modifications reduce H_env_, thereby decreasing the need for stereotyped regulatory behaviours, and they also lower exposure to high−impact sensory input by reducing σ_s_ through sensory accommodations. Predictability−enhancing strategies reduce P(t), the magnitude of prediction error, through tools such as visual schedules, social narratives, and structured routines. Approaches that build uncertainty tolerance target π_k_, using cognitive−behavioural methods designed to address intolerance of uncertainty. Social−motivation−enhancing strategies increase M by identifying and cultivating intrinsically rewarding social contexts. This parameter−level specificity moves beyond generic intervention design and instead supports targeted approaches aligned with individual neurobiological profiles.

#### Neurodiversity−affirming perspective

A central feature of this framework is its reframing of many autistic traits as adaptive responses to neurobiological differences rather than deficits or pathology. Stereotypies are understood as functional mechanisms for entropy reduction and arousal regulation. Restricted interests provide reliable sources of reward, competence, and reduced uncertainty. Insistence on sameness minimises prediction errors in a system characterised by atypical precision−weighting. Social withdrawal conserves executive resources in the context of inefficient long−range connectivity. This perspective emphasises the functional logic of autistic behaviours and supports interventions that respect and work with these adaptive processes.

This perspective aligns with neurodiversity movements and emphasises supporting adaptive strategies whilst reducing environmental demands rather than attempting to “normalise” autistic people through behaviour modification (97, 98).

#### Relationship to existing models

Several computational approaches to autism have been proposed in the literature, and our framework differs from them in several important respects. Compared to machine−learning classification models, this approach prioritises interpretability over classification accuracy, ensuring that every parameter carries a clear mechanistic meaning. In contrast to pure Bayesian models (32, 42) the framework extends beyond social cognition to encompass all DSM−5 symptom domains and incorporates explicit temporal dynamics. Relative to developmental trajectory models (26, 27), it provides mechanistic explanations for why developmental patterns unfold in the ways observed empirically.

By integrating the three major theoretical frameworks of Predictive Coding, Information Theory, and Network Neuroscience within a unified mathematical structure, this model offers a novel contribution that bridges computational neuroscience with clinical phenomenology.

### Integrative vignette: a noisy social gathering

To illustrate how the coupled submodels produce observable behavioural trajectories, we present a hypothetical scenario in which an autistic adult attends a two-hour social event in a busy restaurant. Illustrative parameter values are drawn from the ranges presented in each submodel.

The person arrives at the restaurant at time t = 0. Ambient noise and visual complexity produce a sensory environment with sigma_s = 0.8 (high sensitivity to auditory stimuli) and delta_s approximately equal to 0.02 (near-absent habituation). Cumulative sensory load P(s) therefore accumulates approximately linearly: P(s) approximately equal to sigma_s multiplied by T = 0.8T. By t = 45 minutes, P_total has risen substantially, contributing to increasing physiological arousal Xi(t).

Social reciprocity R(t) begins at R_0 = 0.85 (good initial engagement). The effective depletion rate beta_eff starts at approximately 0.3 per hour, modulated by moderate executive load (E = 0.4), rising sensory load (Lambda_s(t) increasing with P_total), and moderate predictive processing load (Pi_load = 0.5 in a semi-familiar group). Social motivation M = 0.6 and interest alignment I = 0.3 partially buffer depletion. By t = 60 minutes, beta_eff has risen to approximately 0.5 per hour owing to accumulated sensory load, and R(t) has declined to approximately 0.45.

Environmental entropy H_env rises as the restaurant fills and conversations overlap (H_env increasing from 3.0 to 5.5 bits). This drives an increase in repetitive movement amplitude Gamma_eff via the Michaelis-Menten relationship. The individual begins subtle hand movements (Gamma_eff approximately equal to 0.4 at t = 30 minutes, rising to 0.7 at t = 60 minutes). These movements serve an entropy-reduction function and partially regulate arousal.

At t = 70 minutes, the group unexpectedly relocates to a different table. This produces a prediction error with magnitude |x_k - x_bar_k| = 0.6 for the seating arrangement routine (pi_k = 4.0, theta_k = 0.3, s_k = 0.9). The deviation substantially exceeds the tolerance threshold, producing S_k approximately equal to 0.85 (near-maximal distress). The combination of elevated P_total, declining R(t), and acute S_k distress creates a compound demand on regulatory resources.

At t = 75 minutes, the person withdraws from group conversation and engages one person in discussion of a restricted interest (I = 0.9, R_int = 3.2 multiplied by R_norm). This temporarily reduces beta_eff (via elevated k_I multiplied by I) and provides intrinsic reward, stabilising R(t) at approximately 0.35 for the next 15 minutes.

However, cumulative sensory load continues to rise (P_total approximately equal to 0.8 multiplied by 90 = 72 at t = 90 minutes). With the individual s regulatory threshold at approximately P_total = 70, this triggers the need for withdrawal. The individual leaves the restaurant at t = 90 minutes, consistent with the model s prediction of unbounded P_total accumulation when delta_s is near zero. Recovery occurs once the sensory stimulus is removed and P_total begins to decay.

This vignette demonstrates how the coupled dynamics across sensory, social, repetitive behaviour, and sameness domains interact to produce the commonly observed trajectory of initial engagement followed by progressive difficulty and eventual withdrawal. Formal simulation with graphical output represents a natural next step for future empirical work.

### Model limitations and future directions

These models necessarily simplify highly complex neurodevelopmental phenomena. Real−world behaviour emerges from continuous interactions between domains, for example, sensory load influencing social reciprocity and such cross−domain effects are only partially represented in the current formulations. Although the parameters are theoretically grounded, empirical estimation requires extensive data, and future work must establish normative ranges as well as patterns of individual variation for each parameter.

The present models also represent snapshots rather than developmental processes. In reality, parameters are likely to change with age, learning, and intervention, suggesting that future extensions should treat parameters as functions of developmental time. Given the substantial heterogeneity within autism, the framework accommodates individual parameter profiles, but some individuals may not conform to these functional forms. Clustering or mixture−modelling approaches may therefore be necessary to identify distinct mechanistic subtypes.

Interactions between domains remain an important area for further development. Although we note, for example, that high P_total_ may increase β_e_ff, explicit mathematical modelling of such cross−domain interactions remains future work. Ultimately, these models require rigorous empirical validation using longitudinal data from autistic individuals across developmental stages.

### Proposed empirical validation approaches

Future research should prioritise empirical testing and refinement of these models through multiple complementary methods. Ecological Momentary Assessment (EMA) offers a way to capture real−time fluctuations in symptoms across natural contexts, providing rich data on temporal dynamics and contextual modulation. Participants would contribute repeated measurements of social engagement, stereotypy frequency, distress levels, and environmental characteristics throughout the day, allowing the models to be tested against lived, moment−to−moment variability.

Longitudinal cohort studies are essential for tracking developmental trajectories from early childhood into adulthood, enabling estimation of how parameters evolve with age, maturation, and intervention. Such cohorts should include both autistic individuals and those at elevated familial risk to capture a broad range of developmental pathways. Experimental manipulation can further refine the models by systematically varying contextual factors such as environmental predictability, sensory load, or social complexity while measuring symptom expression to isolate the influence of specific parameters.

Neuroimaging approaches provide an additional layer of validation by correlating parameter estimates with neural measures, such as connectivity strength for β_(_b_ase)_, GABAergic function for δ_s_, or striatal activation for reward−related parameters. Intervention studies can then test whether parameter−targeted approaches produce the predicted changes in symptom expression—for example, whether increasing environmental predictability reduces π_k_, or whether executive−function training reduces β_(_b_ase)_.

Robust parameter estimation will require advanced analytical methods, including nonlinear least−squares fitting, hierarchical Bayesian modelling, or state−space models capable of capturing both individual−specific and group−level dynamics from longitudinal data. Cross−validation procedures can then assess whether estimated parameters successfully predict symptom expression in held−out contexts or time periods, providing a stringent test of model generalisability.

### Parameter identifiability and data requirements

The framework proposes a substantial number of person-specific parameters. A legitimate concern is whether these parameters are identifiable from typical datasets. We classify parameters into four tiers based on the data source required for estimation (**Table 5**).

**Table 5:** Parameter identifiability and data requirements.

Tier	Data source	Parameters	Example methods
Tier 1: EMA-identifiable	Repeated self-report or actigraphy in naturalistic settings	beta_eff, M(t) oscillation frequency omega, S_k distress responses, I(t) engagement levels, R(t) trajectory	Smartphone-based EMA; wrist actigraphy; daily diary
Tier 2: Structured observation / lab tasks	Standardised assessments or experimental paradigms	eta_i (channel efficiencies), R_sal (reward salience), theta_k (tolerance thresholds), w_i (channel weights)	ADOS-2 interaction tasks; reward-learning paradigms; change-detection tasks
Tier 3: Neuroimaging-informed	Neural measures correlated with specific parameters	delta_s (habituation rate, linked to GABAergic function), beta_base (linked to long-range connectivity), pi_k (estimable from mismatch negativity)	fMRI connectivity analysis; MRS GABA quantification; EEG mismatch negativity
Tier 4: Population priors	Hierarchical Bayesian estimation with informative priors	GABA_func/GABA_norm ratios, connectivity-cost coefficients, sigma_s absolute scaling	Meta-analytic neuroimaging priors; hierarchical Bayesian modelling of group data

Importantly, not all parameters need to be estimated simultaneously. Given the hierarchical separation of time scales, parameters within each submodel can be estimated independently before cross-model coupling is assessed, substantially reducing the dimensionality of the estimation problem. Nonlinear least-squares fitting, hierarchical Bayesian modelling, and state-space models are all appropriate analytical methods for parameter recovery from longitudinal data.

### Extensions and future theoretical development

Several extensions would substantially strengthen the framework. One important direction is multi−level modelling, in which hierarchical relationships are explicitly represented across neural mechanisms such as connectivity patterns and neurotransmitter function computational processes including prediction errors and entropy, and the behavioural symptoms that emerge from these interactions. Another extension involves adding explicit interaction terms to capture how domains influence one another; for example, expressions such as ∂β_e_ff/∂P_total_ would formalise how increases in sensory load can amplify social fatigue.

A further refinement would be to model parameters themselves as developmental functions, such as β_(_b_ase)_(age), allowing the framework to characterise how mechanisms evolve across the lifespan. Intervention effects could also be formalised mathematically, for instance, representing dπ_k_/dt as a function of uncertainty−tolerance training to capture how precision−weighting changes over time.

The framework could be expanded to incorporate co−occurring conditions, including anxiety, attentional differences, and motor coordination challenges, which frequently shape autistic experience. It would also benefit from modelling compensatory mechanisms, capturing how individuals develop strategies that alter observable symptoms even when underlying mechanisms remain unchanged. Finally, applying similar mechanistic formulations to other neurodevelopmental conditions would allow systematic comparison, helping identify both shared processes and distinct mechanistic signatures.

## Conclusions

This paper presents a mathematical framework for ASD that integrates dynamic systems theory with mechanistic neurobiological principles from Predictive Coding, Information Theory, and Network Neuroscience. By modelling social reciprocity as executive resource depletion, nonverbal communication as multi-channel integration, relationship development as reward-modulated learning, repetitive behaviours as entropy-reduction cycles, insistence on sameness as high-precision prediction error responses, restricted interests as domains of heightened reward salience and prediction accuracy, and sensory sensitivities as habituation failure with cumulative load, we provide an interpretable, quantitative framework for the complex, dynamic nature of autism.

The primary contribution is the explicit derivation of mathematical formulations from mechanistic theories, moving beyond description to explanation. This provides a quantitative language for autistic experience that respects internal consistency and adaptive logic whilst identifying specific neurobiological parameters as intervention targets. The framework enables personalised computational phenotyping, where people are characterised by parameter profiles rather than categorical diagnoses, facilitating precision medicine approaches.

Importantly, by conceptualising many autistic traits as functional adaptations to neurobiological differences, the framework adopts a neurodiversity-affirming perspective. Stereotypies are not behaviours to be extinguished but regulatory mechanisms to be supported. Restricted interests are not pathological fixations but reliable sources of reward and competence. Insistence on sameness is not irrational rigidity but a rational response to aberrant precision-weighting.

The integration of mechanistic theory with dynamic modelling moves the field closer to a true computational phenotype of ASD, one that captures individual differences, developmental trajectories, contextual modulation, and underlying mechanisms within a unified mathematical framework. This approach has the potential to transform both scientific understanding and clinical practice, enabling more precise diagnosis, more targeted intervention, and ultimately improved outcomes and quality of life for autistic people.

Further empirical validation through ecological momentary assessment, longitudinal cohort studies, experimental manipulation, neuroimaging correlation, and intervention trials is warranted to refine parameter estimates, test predictions, and establish clinical utility. The framework provides a foundation for ongoing theoretical development, with clear pathways for extension to developmental change, cross-domain interactions, co-occurring conditions, and intervention effects.

## Data Availability

The original contributions presented in the study are included in the article/supplementary material. Further inquiries can be directed to the corresponding author.
